# Effects of Caffeine and Acute Aerobic Exercise on Working Memory and Caffeine Withdrawal

**DOI:** 10.1038/s41598-019-56251-y

**Published:** 2019-12-23

**Authors:** Anisa Morava, Matthew James Fagan, Harry Prapavessis

**Affiliations:** 1 Western University, Exercise and Health Psychology Lab, Department of Kinesiology, Arthur and Sonia Labatt Health Sciences Building, London, Ontario, N6A 5B9 Canada; 2 University of British Columbia, Population Physical Activity Lab, School of Kinesiology, Lower Mall Research Station, British Columbia, V6T 1Z4 Canada

**Keywords:** Human behaviour, Cognitive neuroscience

## Abstract

Studies show that a single bout of exercise confers cognitive benefits. However, many individuals use psychoactive substances such as caffeine to enhance cognitive performance. The effects of acute exercise in comparison to caffeine on cognition remain unknown. Furthermore, caffeine use is associated with withdrawal symptoms upon cessation. Whether acute exercise can reduce withdrawal symptoms also remains unknown. The objectives of this study were to compare the effects of acute moderate intensity aerobic exercise to caffeine on working memory (WM) and caffeine withdrawal symptoms (CWS). In Phase I, non-caffeine (n = 29) and caffeine consumers (n = 30) completed a WM assessment, followed by acute exercise and caffeine. In Phase II, caffeine consumers (n = 25) from Phase I underwent the WM assessment and reported CWS following a 12-hour deprivation period. Acute moderate intensity aerobic exercise and caffeine (1.2 mg/kg) significantly improved WM accuracy and reduced CWS comparably. WM performance was not reduced following caffeine deprivation.

## Introduction

Cognitive functions are critical for navigating everyday life challenges^1^. Previous work has demonstrated a single bout of exercise has been shown to improve cognitive functions^2–4^. In a comprehensive meta-analysis conducted by Chang *et al*.^2^, the authors found acute exercise (aerobic, anaerobic, resistance, and combination) had a small (Hedge’s *g* = 0.097), but positive effect on cognition. Furthermore, these positive cognitive effects were found during exercise, immediately following exercise, and after a delay^2^. Although acute exercise elicits cognitive enhancements, individuals often utilize psychoactive substances to improve cognition. Caffeine (1, 3, 7-trimethylxanthine) is one of the most widely used psychoactive substances worldwide^5^. The cognitive and mood-enhancing benefits of caffeine have been cited as one of the primary motivators for its consumption^6,7^. Caffeine elicits improvements to multiple cognitive domains including information processing, attention, and specific types of memory^8–12^. Although caffeine elicits benefits to cognitive domains, concerns associated with increased anxiety/anxiety-like symptoms^13,14^, muscle tremors^15,16^, and withdrawal symptoms^17,18^ are present.

Withdrawal symptoms in particular are experienced by a large proportion of caffeine consumers^17^. Caffeine withdrawal symptoms include headache, fatigue, decreased contentedness, and decreased alertness upon cessation^17^. Furthermore, caffeine withdrawal has been associated with reduced cognitive performance^18–20^. Caffeine administration has been shown to reverse caffeine withdrawal symptoms^8^. Interestingly, in other substance use contexts, namely nicotine, acute exercise has been used to reduce the intensity and frequency of withdrawal symptoms and cravings^21,22^. In two comprehensive systematic and meta-analysis reviews, Roberts *et al*.^22^, using aggregate data and Haasova *et al*.^21^, using individual participant data found weighted mean differences in both “desire to smoke” [−1.90 and −2.04 points, respectively] and “strength of desire to smoke” [−2.41 and −1.91 points, respectively] that favoured the acute exercise condition over the control condition following a temporary period of abstinence. The effect sizes found in these studies ranged from d = 0.4 to 1.9, which are considered moderate-to-large in size^23^. Furthermore, craving reduction effects lasted up to 30 minutes post-exercise^24^. Although the mechanisms behind the post-exercise craving and withdrawal symptom reduction remain to be elucidated, the shared symptomatology lends to assessing the utility of acute exercise in reducing caffeine withdrawal symptoms.

Limited studies have examined the effects of acute exercise and caffeine intake on cognition concurrently^25^, however none to our knowledge have examined acute exercise in comparison to caffeine administration on either cognition or caffeine withdrawal symptoms. By comparing acute exercise directly to caffeine administration, which has well-established mechanistic pathways (i.e., antagonism of adenosine receptors), the mechanisms underlying acute exercise-induced benefits to cognitive function can be further elucidated. Thus, the objectives of the present study were to compare acute aerobic exercise to caffeine administration on cognitive performance and caffeine withdrawal symptoms. The first objective (Phase I) was to determine the effects of an acute bout of moderate intensity aerobic exercise and caffeine administration on WM in both non-caffeine and caffeine consumers. The second objective (Phase II) was to determine whether an acute bout of moderate intensity aerobic exercise and caffeine administration could reduce caffeine withdrawal symptoms and restore WM performance after a 12- hour caffeine deprivation period among the caffeine consumers used in Phase I. It was hypothesized in Phase I that in comparison to baseline WM performance, aerobic exercise and caffeine administration would improve WM comparably in both non-caffeine and caffeine consumers. In Phase II, it was hypothesized that aerobic exercise or caffeine administration would reduce caffeine withdrawal symptoms and restore WM performance comparably following a 12-hour caffeine deprivation period.

## Results

### Phase I

#### Non-caffeine consumers

**Figure 1:**
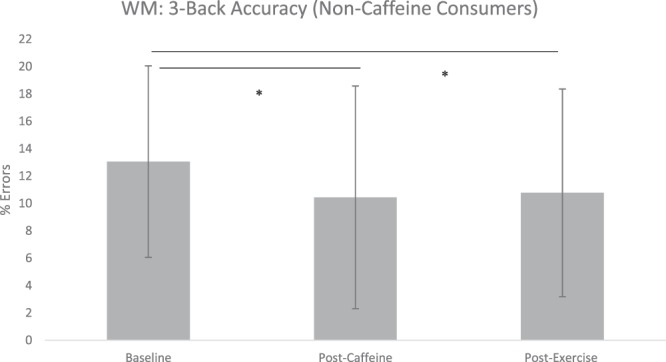
Changes to working memory accuracy (% errors) on the 3-back load following caffeine and exercise treatments in non-caffeine consumers. Values are means ± SD. *p < 0.05 ^†^WM = working memory.

A repeated measures ANOVA for 3-back accuracy was statistically significant: F(2, 56) = 3.315, *p* = 0.044, η^2^ = 0.106 (Fig. 1). Paired sample post-hoc t-tests uncovered non-significant differences between baseline and the caffeine condition: t(28) = 2.60, p = 0.052, d = 0.345, baseline and the exercise condition: t(28) = 2.30, *p* = 0.107, d = 0.313, and caffeine and exercise condition t(28) = 0.25, *p* = 1.000, d = 0.0148. A repeated measures ANOVA for 3-back RT was not statistically significant: F(2, 56) = 1.233, *p* = 0.299, η^2^ = 0.042.

#### Caffeine consumers

**Figure 2:**
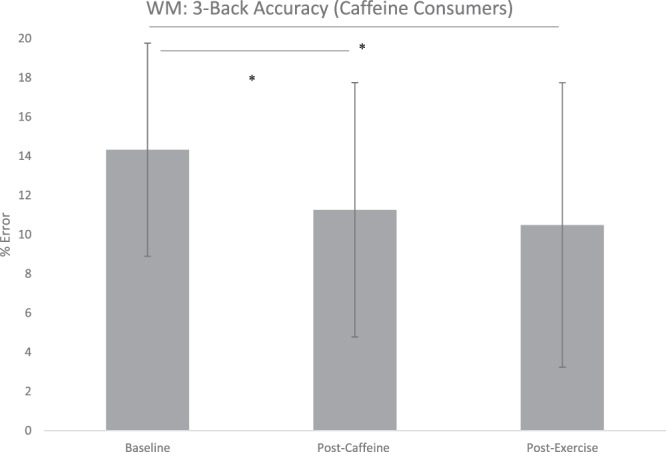
Changes to working memory accuracy (% errors) on the 3-back load following caffeine and exercise treatments in caffeine consumers. Values are means ± SD. *p < 0.05 ^†^WM = working memory.

A repeated measures ANOVA for 3-back accuracy was statistically significant: F(2, 58) = 6.479, p = 0.003, η^2^ = 0.183 (Fig. 2). Paired sample post-hoc t-tests uncovered significant differences between baseline and the caffeine condition: t(29) = 2.818, p = 0.027, d = 0.512, and baseline and the exercise condition: t(29) = 3.454, p = 0.006, d = 0.599. No significant difference was found between the caffeine and exercise condition t(29) = 0.667, p = 1.000, d = 0.112. A repeated measures ANOVA for 3-back RT was not statistically significant: F(2, 58) = 1.157, p = 0.321, η^2^ = 0.038.

### Phase II

#### Caffeine withdrawal symptoms

**Figure 3:**
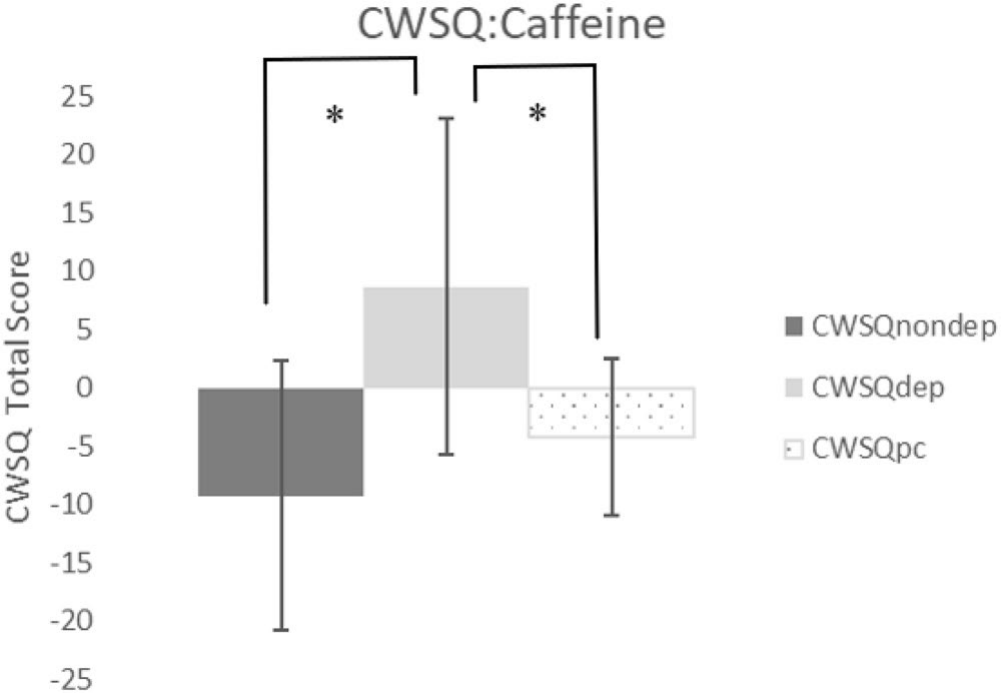
Changes to caffeine withdrawal symptoms from the non-deprived state, following 12-hour deprivation, and post caffeine administration. Values are means ± SD. *p < 0.05. ^†^CWSQ = caffeine withdrawal symptom questionnaire. ^††^Non-dep = non-caffeine deprived, dep = caffeine deprived, pc = post caffeine.

A repeated measures ANOVA between non-deprived CWSQ, deprived CWSQ, and post-caffeine CWSQ scores was statistically significant: F(2, 24) = 11.058, p = 0.001, η^2^ = 0.501 (Fig. 3). Paired sample post-hoc t-tests uncovered significant differences between baseline and the deprived condition, t(11) = −3.856, p = 0.008, d = 1.35, as well as between the deprived condition and post-caffeine administration: t(11) = −3.392, p = 0.018, d = 1.15.

**Figure 4:**
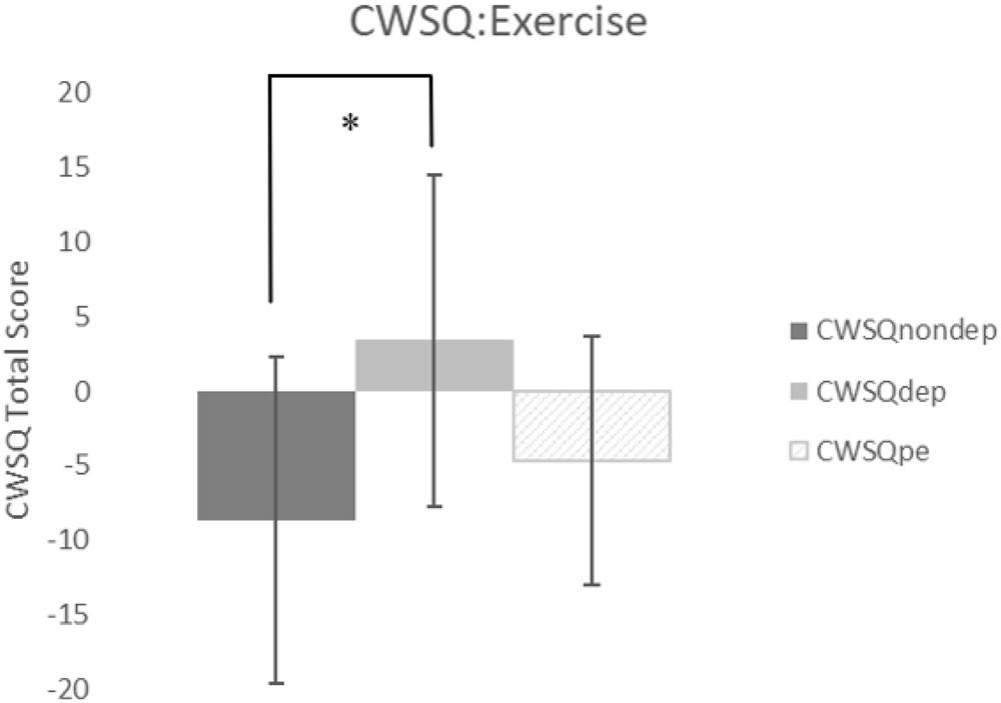
Changes to caffeine withdrawal symptoms from the non-deprived state, following 12-hour deprivation, and post exercise administration. Values are means ± SD. *p < 0.05. ^†^CWSQ = caffeine withdrawal symptom questionnaire. ^††^Non-dep = non-caffeine deprived, dep = caffeine deprived, pe = post exercise.

A repeated measures ANOVA between non-deprived CWSQ, deprived CWSQ, and post-exercise CWSQ scores was also statistically significant: F(2, 24) = 5.786, p = 0.009 η^2^ = 0.325 (Fig. 4). Paired sample post-hoc t-tests uncovered a significant difference between baseline and the deprived condition, t(12) = −2.861, p = 0.043, d = 1.095, but a non-significant difference between the deprived condition and post exercise t(12) = −1.338, p = 0.062, d = 0.730.

### Caffeine withdrawal and WM

Repeated measures ANOVAs for 3-back accuracy and RT between non-deprived, deprived, and post-caffeine WM performance were not statistically significant, respectively: F(2, 22) = 0.651, p = 0.531, η^2^ = 0.056; F(2, 22) = 0.684, p = 0.515, η^2^ = 0.059. Repeated measures ANOVAs for 3-back accuracy and RT between non-deprived, deprived and post-exercise WM performance were also not statistically significant, respectively: F(2, 24) = 1.801, p = 0.187, η^2^ = 0.131; F(2, 24) = 0.486, p = 0.621, η^2^ = 0.039.

## Discussion

Results from the present study indicate that acute aerobic exercise and caffeine administration improved WM accuracy in non-caffeine and caffeine consumers on the most difficult load of the n-back task (3-back). Furthermore, acute aerobic exercise and caffeine administration demonstrated some utility in reducing caffeine withdrawal symptoms induced by a 12-hour caffeine deprivation period. Interestingly, no decrements to WM were detected following a 12-hour caffeine deprivation period. Beyond these overarching findings, several issues warrant further discussion.

In Phase I, acute aerobic exercise and caffeine administration conferred comparable improvements to accuracy (absolute percent difference: 2.62%, 2.29% and relative percent difference: 20.1%, 17.5% respectively). For non-caffeine consumers, caffeine administration conferring a marginal accuracy benefit may be due in part to the novelty of caffeine as a substance. Prior research has suggested non-caffeine consumers display heightened physiological and psychological responses to caffeine^26^. Furthermore, the non-caffeine consumers in this study reported high physical activity participation (Table 1), suggesting tolerance of a single-bout of aerobic exercise with little fatigue and discomfort^27^. Previous studies have identified that exercise tolerance is implicated in exercise-cognition investigations as individuals who do not regularly exercise are more likely to experience fatigue, which has been associated with impaired cognitive performance^28^. It is also important to note that our findings contribute to the body of literature^8,14^ supporting the notion that caffeine provides net benefits to cognition and does not rely completely on the reversal of withdrawal symptoms, as non-caffeine consumers would not be expected to experience caffeine withdrawal.

**Table 1:** Values represent means and standard deviations (SD) of demographic variables.

	Caffeine Consumers (n = 30)	Non-Caffeine Consumers (n = 29)
Age (years)	24.1 (4.8)	24.8 (3.4)
Sex (% males)	43.3%	51.7%
Weight (kg)	72.7 (15.1)	70.1(12.2)
**Education (%)**		
Undergraduate	50.0%	13.33%
Graduate	43.3%	86.67%
Employed	6.67%	0%
**Caffeine Intake (mg)**		
Weekly	2110.2(1194.8)	74.7 (64.4)
Daily	301.5 (170.7)	10.7 (9.8)
Time of Last Caffeine Consumption (h)	10.33 (9.3)	
Years of Caffeine Consumption	6.7 (4.1)	
Preferred Type of Caffeine Administration	Coffee	
Physical Activity (minutes of MVPA/week)	1213 (752.8)	1324.19 (1044.3)

h = hours, mg = milligrams, h = hours, MVPA = moderate-to-vigorous physical activity.

In caffeine consumers, acute aerobic exercise improved accuracy to a greater extent (absolute percent difference: 3.84%, relative percent difference: 26.8%) than caffeine administration (absolute percent difference: 3.07%, relative percent difference: 21.4%). Aerobic exercise conferring a greater benefit to WM accuracy than caffeine may be due in part to caffeine tolerance^29^. The caffeine dose administered (1.2 mg/kg) equates to less than the mean daily caffeine consumption reported by the caffeine group (301.5 mg/day), suggesting these consumers have likely developed some level of tolerance to the caffeine-driven cognitive effects. Similarly, to the non-caffeine consumers, caffeine consumers also reported regular participation in physical activity (Table 1) supporting the notion that a single-bout of aerobic exercise was tolerated comfortably by this group.

Our investigation did not find improvements to WM speed (RT) as a result of acute aerobic exercise or caffeine administration in both non-caffeine and caffeine consumers on the 3-back load. These findings differ from those reported by Haskell *et al*.^30^, and McMorris *et al*.^31^. Diverging results could be due to the wide range in administered caffeine doses^32^, type of cognitive task administered, and exercise intensity^12,31^. Prior work by our group also detected no changes to RT on the n-back task following acute aerobic exercise at a moderate intensity (Fagan *et al*., unpublished). It is important to note when examining the WM speed and accuracy findings in concert, improved WM was not due to a speed-accuracy trade-off^33^. In other words, individuals were not committing less errors on the n-back task at a cost to response speed. Prior work has suggested caffeine may improve accuracy in cognitive tasks via increased alertness^34^ and modulation of neuronal activity in regions associated with attention^35^. When considering acute aerobic exercise it has been proposed that exercise selectively affects the activation and allocation of attentional resources^4,36^. Thus, the improved WM accuracy that was observed may be in part due to increased general arousal. A battery of cognitive tests could have aided in elucidating whether the effects were WM-specific or a reflection of global cognitive improvement.

It is important to address that a treatment by order effect was detected for accuracy on the 3-back load in caffeine consumers, suggesting receiving caffeine on the first day may have resulted in improved performance on the second day following acute aerobic exercise, although treatment order was counterbalanced. A carry-over effect may have been present and thus utilizing a wash-out period greater than 24-hours and employing a between-groups placebo design may be required in future investigations.

In Phase II, a twelve-hour caffeine deprivation period increased subjective caffeine withdrawal symptoms (14.88-point increase on CWSQ from non-deprived state), which was in line with prior work examining caffeine withdrawal^17^. Moreover, caffeine administration and aerobic exercise reduced caffeine withdrawal symptoms (12.91-point reduction, 8.07 point-reduction, respectively). Our results support previous work that suggest caffeine re-administration reduces caffeine withdrawal symptoms^8^. Furthermore, our study suggests acute aerobic exercise demonstrates some utility in reducing caffeine withdrawal symptoms, which is a novel finding, as well as provides further evidence that a single-bout of aerobic exercise improves “alertness”, “feelings of energy”, and mood^36,37^. In addition, our findings are consistent with work conducted in the exercise and tobacco withdrawal literature, which showed acute aerobic exercise successfully reduced withdrawal symptoms such as stress, difficulty concentrating, tension, restlessness, depression, and irritability^22^.

In contrast to the caffeine withdrawal symptoms, a 12-hour caffeine deprivation period did not reduce WM performance in caffeine consumers. No significant changes to WM accuracy or speed were detected between the non-caffeine deprived and caffeine-deprived conditions. These findings were not in line with work conducted by Yeomans *et al*.^20^. Differing results may be due to the duration of caffeine-deprivation utilized in our paradigm. Some studies have employed a 24-hour caffeine deprivation period which may have resulted in greater caffeine withdrawal severity and in turn greater cognitive deficits^20,38^. In addition, depending on the caffeine consumption pattern of the individual, the 12-hour overnight deprivation period may have been sufficient to induce withdrawal symptoms in individuals who consume caffeine in the early morning, but not those in the early afternoon.

Furthermore, when considering the cognitive tasks that were administered in the investigations of caffeine withdrawal that detected a caffeine-deprivation induced cognitive deficit, a variety of cognitive tasks were used (e.g., Rapid Visual Information Processing task, Attention Network Test) and thus perhaps, the n-back alone may not have been the most sensitive to detect subtle WM deficits^39^. Alternatively, the caffeine consumers in our study completed several iterations of the n-back task, thus the practice effect may have bolstered WM performance in the caffeine-deprived trials; however other investigations have suggested the stability of performance on higher loads of the n-back suggest a limited role of practice^40^.

Addicott and Laurienti^8^ have also posited participants may exert more effort during the caffeine-deprived state to compensate for “withdrawal-related fatigue”. Given that WM performance did not suffer following the 12-hour deprivation period, improvement to WM via caffeine administration or acute aerobic exercise was unlikely. However, it is important to note that WM performance remained stable following both treatments. Previous literature has suggested that caffeine withdrawal effects worsen with time and withdrawal related fatigue could result in deteriorating performance on cognitive tasks^17,39^. Thus, since we detected no change to WM performance, the caffeine administration and acute aerobic exercise treatments may have buffered the caffeine-deprivation effects.

In this investigation, a single dose of caffeine (1.2 mg/kg) and a single bout of moderate intensity exercise was examined. Future investigations should examine varying caffeine doses and exercise intensities to establish dose-response curves. Another important future direction is to determine the duration of caffeine and exercise effects on cognitive performance. Prior work has suggested depending on the vehicle of caffeine administration and cognitive assessment employed, caffeine has demonstrated cognitive effects lasting between 1–5 hours^41^. In regards to acute exercise, the duration of cognitive effects post-exercise remains an area of investigation^42^. Furthermore, an important variable to examine in conjunction with caffeine, exercise, and cognition is sleep. Future work should collect and examine sleep-related variables including sleep duration, sleep quality, chronotype, and homeostatic sleep pressure.

Findings from the present study suggest acute aerobic exercise and caffeine administration improve WM accuracy in both non-caffeine and caffeine consumers comparably. Furthermore, caffeine administration significantly reduced caffeine withdrawal symptoms induced by a 12-hour deprivation period, while acute aerobic exercise reduced caffeine withdrawal symptoms, albeit not significantly. WM was not reduced following caffeine deprivation, hence whether exercise and caffeine can restore WM remains unknown. Through investigations of this nature, the utility of acute aerobic exercise in lieu of caffeine consumption to optimize cognitive performance and reduce caffeine withdrawal symptoms would be further clarified with the end-goal of guiding health-related interventions for both general and special populations.

## Methods

### Participants

Twenty-nine non-caffeine consumers (<30 mg caffeine/day^30^) and thirty caffeine consumers (≥150 mg/day^40^) aged 18–40 participated in Phase I of the study. Twenty-five of the caffeine consumers in Phase I participated in Phase II of the study. Participants were excluded if they displayed any contraindications to exercise (as assessed by the Physical Activity Readiness Questionnaire; PAR-Q), reported cognitive difficulties, reported taking of medication for depression or anxiety, or were pregnant. Prior to participation, each participant read a letter of information outlining all study procedures, as well as potential risks and benefits associated with participation, which was approved by the Western University Research Ethics Board. All study procedures were carried out in accordance with the revised version of the Helsinki Declaration (2013). Informed consent was obtained from all study participants.

### Study design

**Figure 5:**
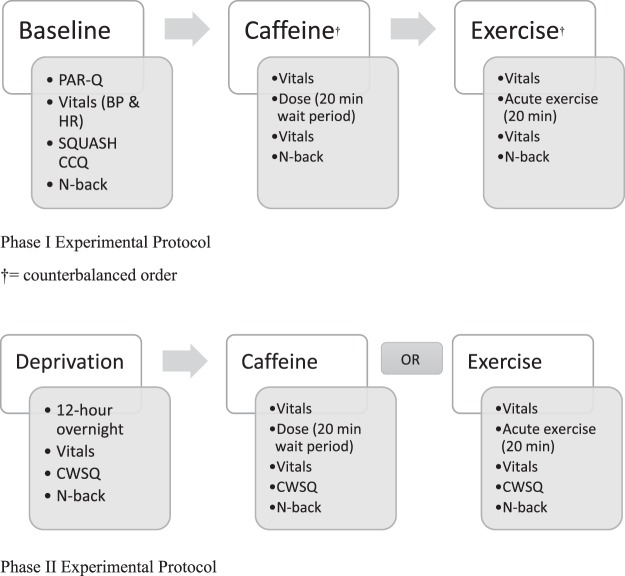
Schematic representation of study protocol.

Phase I utilized a randomized counterbalanced crossover design such that each participant was randomly assigned treatment order (i.e., caffeine administration followed by exercise or exercise followed by caffeine administration) but completed both treatments irrespective of being non-caffeine and caffeine consumers (Fig. 5). Phase II involved only caffeine consumers and utilized a randomized design such that each participant was randomly assigned to receiving either caffeine administration or exercise following a 12-hour caffeine deprivation period (Fig. 5). Randomization was completed using a computer-generated numbers table.

### Sample size

#### Phase I

Giles and colleagues^38^ detected a change in WM accuracy (composite score of hit rate and false alarm rate) between placebo and caffeine administration (Cohen’s *d* = 0.418). Fagan, Guirguis, Smith, Sui, Rollo, and Prapavessis, unpublished detected a change in WM accuracy (% errors) between baseline and aerobic exercise (Cohen’s *d* = 0.511). Based on the above findings, to be adequately powered to detect differences from baseline, caffeine, and aerobic exercise, a conservative approach of using a small-to-moderate effect size f = 0.20, power = 0.80, and alpha = 0.05, generated a sample size of 28 individuals^23,43^.

#### Phase II

In developing the Caffeine Withdrawal Symptom Questionnaire (CWSQ), Juliano and colleagues^44^ detected a 2.69-point reduction in withdrawal symptoms (Cohen’s *d* = 0.866) when caffeine was administered following a 16-hour caffeine deprivation period. Based on the above findings, to be adequately powered to detect the effects of caffeine administration following an overnight deprivation period, an approach of using the effect size of *d* = 0.866, power = 0.80, and alpha = 0.05, generated a sample size of 13 individuals^23,43^.

### Measures

#### Demographics

Age, sex, weight (kg), and education level were collected (Table 1).

### Caffeine and drug consumption history

Acute and chronic caffeine history (i.e., time of last caffeine consumption, amount of years regularly consuming caffeine, preferred type of caffeine administration) was assessed. Drug and alcohol consumption in the past 18 hours were also assessed (Table 1).

### Physical activity

The Physical Activity Readiness Questionnaire (PAR-Q)^45^ was utilized to assess ability to participate in physical activity safely. The PAR-Q is appropriate to administer to individuals aged 15–69 years^45^.

The Short Questionnaire to Assess Health-enhancing Physical Activity (SQUASH)^46^ was administered to assess the frequency, duration, and perceived effort of physical activity during an average week in four domains: commuting (e.g. walking to school), leisure time (e.g. sports), household (e.g. washing dishes), and work/school (e.g. walking and standing between working at a desk)^46^. Frequency and duration are fillable options, such that the participant is able to indicate the number of days per week, as well as the amount of hours and minutes they partake in each activity, while perceived effort has three possible options: slow/light, moderate, and fast/intense.

### Caffeine consumption

The Caffeine Consumption Questionnaire Revised (CCQ-R)^47^ was administered to assess the consumption of caffeine-containing products (i.e., beverages, foods, and drugs) during an average week. The CCQ-R provides images of caffeine containing products to aid in the estimation of the serving size of products consumed. CCQ-R responses were converted to caffeine intake in milligrams/week using the reference values in Harland^40^.

### Working memory

Working memory (WM) was assessed through the n-back task. The n-back task has been widely used in the cognition literature to gauge WM, as it requires both short-term recognition of and operation on stimuli^48,49^. The n-back task consists of a series of stimuli that are presented rapidly on a screen, with the participant deciding whether the target stimuli matches the stimuli ‘n’ items back^50^. Participants would complete each load (0-back, 1-back, 2-back, and 3-back) three times in a randomized order. The 3-back load is the most cognitively demanding and has been shown to be most sensitive to drug effects^51^.

### Caffeine withdrawal

The Caffeine Withdrawal Symptom Questionnaire (CWSQ)^44^ was utilized to assess the type and severity of caffeine withdrawal symptoms experienced by the caffeine consumers. The CWSQ uses twenty-three items which focus on seven symptom clusters: (1) fatigue/drowsiness, (2) low alertness/difficulty concentrating, (3) mood disturbances, (4) low sociability/motivation to work, (5) nausea/upset stomach, (6) flu-like feelings, and (7) headache. The CWSQ also includes nine additional items for consideration, four of which have not yet been empirically validated. Severity of each symptom is assessed on a five-point scale ranging from 0 (*not at all*) to 4 *(extremely*). A higher score reflects greater number of symptoms and symptom severity.

### Interventions

#### Aerobic exercise

The exercise intervention consisted of a single bout of moderate intensity aerobic exercise completed on a Woodway PPS treadmill (Woodway, Waukesh, WI). The intervention consisted of a 2.5 minute warm-up walk, 15 minutes walking at a moderate intensity, and a 2.5 minute cool-down walk. Moderate intensity exercise was defined as 40 to 60% of Heart Rate Reserve (HRR)^52,53^. The researcher controlled the speed and incline of the treadmill to ensure the participant exercised within their moderate intensity HRR range.

### Caffeine administration

The caffeine administration intervention consisted of oral ingestion of powdered caffeine. Each participant ingested 1.2 mg/kg (body weight) of powdered caffeine (Sigma–Aldrich Foundation, St Louis, MO) dissolved in 100 mL of water^39^. The participant then waited in a seated position for 20 minutes to permit caffeine absorption^53^.

### Procedures

Participants were initially screened for eligibility via email or an in-person meeting. For those eligible, a first session was scheduled at the Exercise and Health Psychology Lab. The first session began with administration of the PAR-Q. If a participant indicated yes to any of the seven items on the PAR-Q, they were deemed not able to participate in physical activity and were thus excluded from the study. Upon completion of the PAR-Q, participants were given the demographic questionnaire, caffeine and drug history questionnaire, SQUASH, CCQ-R, and the CWSQ (caffeine consumers only) to complete. A non-caffeine consumer was defined as an individual who consumes less than 30 mg of caffeine/day^26^. A caffeine consumer was defined as an individual who consumes equal to or greater than 150 milligrams of caffeine a day, which approximately equates to the amount of caffeine in a cup of brewed coffee^40^. Blood pressure (BP) was taken in a seated position with an electronic sphygmomanometer (MPOW). Resting heart rate (HR) was taken in a seated position with a heart rate monitor (Polar RS100). Weight was measured using the Health-O-Meter Professional weight scale (Health-O-Meter 500 KL, Boca Ration, FL) to the nearest 0.1 kg. Participants then completed the baseline n-back task (lasting approximately 10 to 15 minutes) on a portable computer in isolation. Participants underwent a practice phase to familiarize themselves with the task. The participant needed to score a minimum of 75% of the trials correctly during the practice phase to proceed to the evaluation. The 75% accuracy threshold was deemed appropriate for mitigating the learning effect on the n-back task in a previous study examining WM in smokers and non-smokers (Fagan, Guirguis, Smith, Sui, Rollo, and Prapavessis, unpublished). Upon completion of the baseline n-back task, participants completed either the aerobic exercise or the caffeine administration. HR and BP were again taken at the end of each intervention followed by the n-back task. In session two, participants underwent the intervention they did not receive on session one. All sessions occurred between 8 a.m. and 5 p.m. and were scheduled at approximately the same time of day (i.e., if the participant came in for their baseline session at 8 a.m. all other sessions occurred at the same time) and were separated by a minimum of 24 hours apart. Regarding caffeine consumption during the assessments in Phase I, caffeine consumers were permitted to consume their regular caffeinated products up to 4 hours prior to their session. For example, if a participant was scheduled for a session at 10 am and they have a single serving of coffee normally every day at 6 a.m. they were permitted to do so. However, if their session was at 10 a.m. and they normally have their single serving of coffee between 7 a.m. and 10 a.m. they were not permitted to do so. This strategy mitigates the caffeine consumers from being partially caffeine-withdrawn before testing^8^.

Caffeine consumers underwent one additional session (Phase II), which required an overnight (12- hour) caffeine deprivation period prior to arrival (i.e., the participant stopped the consumption of any caffeinated products at 8 p.m. and had to come into the lab at 8 a.m.). Participants were told the researcher would be biologically confirming caffeine abstinence through a saliva swab, when in fact no salivary caffeine assays were conducted. This was simply a strategy to increase caffeine deprivation compliance^54^. Participants’ BP and HR were taken in a seated position upon arrival. They then completed the CWSQ and the n-back task to assess caffeine-deprived performance. Upon completion of the n-back task, participants were randomized into receiving either the exercise session or caffeine administration session. At the end of either session, the CWSQ and n-back were administered again. At the end of the experimental protocols, participants’ email addresses were entered into a draw to win a twenty-five-dollar gift card.

### Statistical analyses

#### Phase I

Repeated measures ANOVAs were conducted across baseline, caffeine, and exercise for both accuracy (% errors) and reaction time (RT) in milliseconds (ms) for non-caffeine and caffeine consumers on the n-back task. Analyses focused on the 3-back load. Descriptive data for two, one, and zero-back load can be found under Supplementary Information.

#### Phase II

For the caffeine consumers assigned to the caffeine session, a repeated measures ANOVA was conducted across baseline (non-caffeine deprived), caffeine deprived (following 12-hour deprivation), and post-caffeine administration on caffeine withdrawal symptom scores. A repeated measures ANOVA was also conducted across baseline (non-caffeine deprived), caffeine deprived (following 12-hour deprivation), and post-caffeine administration on accuracy and RT on the n-back task. For the caffeine consumers assigned to the exercise session, identical analyses as listed above were conducted. Analyses focused on the 3-back load. Descriptive data for the two, one, and zero-back load can be found under Supplementary Information.

For both phases, all data were assessed for normality (Shapiro-Wilks) and following significant repeated measures ANOVAs, Bonferroni-corrected post-hoc t-tests were conducted. The level of significance was accepted at *p* ≤ 0.05 for all tests. Effect sizes (Cohen’s *d*, η^2^) accompany all reported findings. All bars in figures represent standard deviation (SD). Data were analyzed using IBM SPSS Statistics (Version 23).

## Supplementary information

N-back

## Data Availability

The datasets generated during and/or analysed during the current study are available from the corresponding author on reasonable request.

## References

[1] Lee MT, Jang Y, Chang WY (2019). How do impairments in cognitive functions affect activities of daily living functions in older adults?. PloS One.

[2] Chang Y-K, Labban JD, Gapin JI, Etnier JL (2012). The effects of acute exercise on cognitive performance: a meta-analysis. Brain Res..

[3] Lambourne K, Tomporowski P (2010). The effect of exercise-induced arousal on cognitive task performance: a meta-regression analysis. Brain Res..

[4] Tomporowski PD (2003). Effects of acute bouts of exercise on cognition. Acta Psychol..

[5] World Health Organization. Psychoactive substances and their sociolegal status. *Neuroscience of Psychoactive Substance Use and Dependence*, https://www.who.int/substance_abuse/publications/en/Neuroscience.pdf (2004).

[6] Temple JL, Dewey AM, Briatico LN (2010). Effects of acute caffeine administration on adolescents. Exp. Clin. Psychopharm..

[7] Yeomans MR (2010). Understanding individual differences in acquired flavour liking in humans. Chemosens. Percept..

[8] Addicott MA, Laurienti PJ (2009). A comparison of the effects of caffeine following abstinence and normal caffeine use. Psychopharmacology.

[9] Fagan D, Swift CG, Tiplady B (1988). Effects of caffeine on vigilance and other performance tests in normal subjects. J. Psychopharmacol.

[10] Fine BJ (1994). Effects of caffeine or diphenhydramine on visual vigilance. Psychopharmacology.

[11] Lieberman HR, Wurtman RJ, Emde GG, Roberts C, Coviella ILG (1987). The effects of low doses of caffeine on human performance and mood. Psychopharmacology.

[12] Smit HJ, Rogers PJ (2005). Effects of low doses of caffeine on cognitive performance, mood and thirst in low and higher caffeine consumers. Psychopharmacology.

[13] Alsene K, Deckert J, Sand P, de Wit H (2003). Association between A 2a receptor gene polymorphisms and caffeine-induced anxiety. Neuropsychopharmacology.

[14] Childs E, de Wit H (2006). Subjective, behavioral, and physiological effects of acute caffeine in light, nondependent caffeine users. Psychopharmacology.

[15] Bovim G, Næss P, Helle J, Sand T (1995). Caffeine influence on the motor steadiness battery in neuropsychological tests. J. Clin. Exp. Neuropsychol..

[16] Sands HR (2015). Mood and psychomotor tremor changes following acute caffeine consumption in moderate and minimal caffeine consumers. J. Caffeine. Res..

[17] Juliano LM, Griffiths RR (2004). A critical review of caffeine withdrawal: empirical validation of symptoms and signs, incidence, severity, and associated features. Psychopharmacology.

[18] James JE (1998). Acute and chronic effects of caffeine on performance, mood, headache, and sleep. Neuropsychobiology.

[19] Lane JD, Phillips-Bute BG (1998). Caffeine deprivation affects vigilance performance and mood. Physiol. Behav..

[20] Yeomans MR, Ripley T, Davies LH, Rusted J, Rogers PJ (2002). Effects of caffeine on performance and mood depend on the level of caffeine abstinence. Psychopharmacology.

[21] Haasova M (2013). The acute effects of physical activity on cigarette cravings: systematic review and meta-analysis with individual participant data. Addiction.

[22] Roberts V, Maddison R, Simpson C, Bullen C, Prapavessis H (2012). The acute effects of exercise on cigarette cravings, withdrawal symptoms, affect, and smoking behaviour: systematic review update and meta-analysis. Psychopharmacology.

[23] Cohen, J. *Statistical Power Analysis for the Behavioral Sciences*. (1988).

[24] Ussher M, Cropley M, Playle S, Mohidin R, West R (2009). Effect of isometric exercise and body scanning on cigarette cravings and withdrawal symptoms. Addiction.

[25] Huertas F, Blasco E, Moratal C, Lupiañez J (2019). Caffeine intake modulates the functioning of the attentional networks depending on consumption habits and acute exercise demands. Scientific reports.

[26] Kennedy DO, Haskell CF (2011). Cerebral blood flow and behavioural effects of caffeine in habitual and non-habitual consumers of caffeine: A near infrared spectroscopy study. Biol. Psychol..

[27] Chiu LZ, Barnes JL (2003). The fitness-fatigue model revisited: Implications for planning short-and long-term training. Strength. Cond. J..

[28] Brown DM, Bray SR (2015). Isometric exercise and cognitive function: an investigation of acute dose–response effects during submaximal fatiguing contractions. J. Sports Sci..

[29] Evans SM, Griffiths RR (1999). Caffeine withdrawal: a parametric analysis of caffeine dosing conditions. J. Pharmacol. Exp. Ther..

[30] Haskell CF, Kennedy DO, Wesnes KA, Scholey AB (2005). Cognitive and mood improvements of caffeine in habitual consumers and habitual non-consumers of caffeine. Psychopharmacology.

[31] McMorris T, Sproule J, Turner A, Hale BJ (2011). Acute, intermediate intensity exercise, and speed and accuracy in working memory tasks: a meta-analytical comparison of effects. Physiol. Behav..

[32] Kaplan GB (1997). Dose-dependent pharmacokinetics and psychomotor effects of caffeine in humans. J. Clin. Pharmacol..

[33] Reed AV (1973). Speed-accuracy trade-off in recognition memory. Science.

[34] Giesbrecht T, Rycroft JA, Rowson MJ, De Bruin EA (2010). The combination of L-theanine and caffeine improves cognitive performance and increases subjective alertness. Nutritional neuroscience.

[35] Koppelstaetter F (2008). Does caffeine modulate verbal working memory processes? An fMRI study. Neuroimage.

[36] Loy BD, O’Connor PJ, Dishman RK (2013). The effect of a single bout of exercise on energy and fatigue states: a systematic review and meta-analysis. Fatigue.

[37] Maraki M (2005). Acute effects of a single exercise class on appetite, energy intake and mood. Is there a time of day effect? Appetite.

[38] Giles GE (2012). Differential cognitive effects of energy drink ingredients: caffeine, taurine, and glucose. Pharmacol. Biochem. Behav..

[39] Heatherley SV, Hayward RC, Seers HE, Rogers PJ (2005). Cognitive and psychomotor performance, mood, and pressor effects of caffeine after 4, 6 and 8 h caffeine abstinence. Psychopharmacology.

[40] Epperson CN, Amin Z, Ruparel K, Gur R, Loughead J (2012). Interactive effects of estrogen and serotonin on brain activation during working memory and affective processing in menopausal women. Psychoneuroendocrinology.

[41] Paulus R (2015). Impact of various caffeine vehicles on mood and cognitive, neurological, and physiological functions over five hours. Ohio J Sci..

[42] Barella LA, Etnier JL, Chang YK (2010). The immediate and delayed effects of an acute bout of exercise on cognitive performance of healthy older adults. J Aging Phys Act.

[43] Faul F, Erdfelder E, Lang AG, Buchner A (2007). G* Power 3: A flexible statistical power analysis program for the social, behavioral, and biomedical sciences. Behavior Research Methods.

[44] Juliano LM, Huntley ED, Harrell PT, Westerman AT (2012). Development of the caffeine withdrawal symptom questionnaire: caffeine withdrawal symptoms cluster into 7 factors. Drug Alcohol Depen..

[45] Thomas S, Reading J, Shephard RJ (1992). Revision of the physical activity readiness questionnaire (PAR-Q). Can. J. Sport Sci..

[46] Wendel-Vos GCW, Schuit AJ, Saris WHM, Kromhout D (2003). Reproducibility and relative validity of the short questionnaire to assess health-enhancing physical activity. J. Clin. Epidemiol..

[47] Irons JG, Bassett DT, Prendergast CO, Landrum RE, Heinz AJ (2016). Development and initial validation of the caffeine consumption questionnaire-revised. J. Caffeine Res..

[48] Baddeley A (1992). Working memory. Science.

[49] Conway ARA (2005). Working memory span tasks: A methodological review and user’s guide. Psychon. Bull. Rev..

[50] Jonides J (1997). Verbal working memory load affects regional brain activation as measured by PET. J. Cogn. Neurosci..

[51] Patterson F (2010). Working memory deficits predict short-term smoking resumption following brief abstinence. Drug Alcohol Dep..

[52] Karvonen, M. J., Kentala, E. & Mustala, O. The effects of training on heart rate. *Ann. Med. Exp. Biol. Fenn*. **35** (1957). 13470504

[53] American College of Sports Medicine. *ACSM’s Guidelines for Exercise Testing and Prescription*. (ed. ACSM) 4-5 (Lippincott Williams & Wilkins, 2013). 10.1249/JSR.0b013e31829a68cf 23851406

[54] Rogers PJ, Martin J, Smith C, Heatherley SV, Smit HJ (2003). Absence of reinforcing, mood and psychomotor performance effects of caffeine in habitual non-consumers of caffeine. Psychopharmacology.

