# Supplementary material for: Effects of Caffeine and Acute Aerobic Exercise on Working Memory and Caffeine Withdrawal
Source: Sci Rep. 2019 Dec 23;9:19644. doi: 10.1038/s41598-019-56251-y (PMC6927973; doi:10.1038/s41598-019-56251-y)
Supplement: Supplementary file 1 — N-back [file 41598_2019_56251_MOESM1_ESM.docx]

Effects of Caffeine and Acute Aerobic Exercise on Working Memory and Caffeine Withdrawal

Anisa Morava MA^1*^, Matthew James Fagan MA^2^, Harry Prapavessis PhD^1^,

^1^ Western University, Exercise and Health Psychology Lab, Department of Kinesiology, Arthur and Sonia Labatt Health Sciences Building, London, Ontario, Canada, N6A 5B9

^2^ University of British Columbia, Population Physical Activity Lab, School of Kinesiology, Lower Mall Research Station, British Columbia, Canada, V6T 1Z4

*Corresponding Author: Anisa Morava, 519-661-2111 ext. 80173, amorava@uwo.ca

Means, standard deviations, and 95% confidence intervals for n-Back task

| Trial | Caffeine Consumers | | |  |
| --- | --- | --- | --- | --- |
|  | *M* | SD | *95% CI* |  |
| Baseline |  |  |  |  |
| 3-back Error % | 14.33 | 5.43 | [12.30, 16.36] |  |
| 3-back RT | 761.12 | 228.71 | [675.72, 846.52] |  |
| 2-back Error % | 8.26 | 7.28 | [5.54, 10.98] |  |
| 2-back RT | 665.80 | 174.99 | [600.45, 731.14] |  |
| 1-back Error % | 5.53 | 7.69 | [2.66, 8.41] |  |
| 1-back RT | 608.62 | 128.98 | [560.45, 656.78] |  |
| 0-back Error % | 4.13 | 7.34 | [1.39, 6.88] |  |
| 0-back RT | 496.29 | 96.54 | [460.24, 532.33] |  |
| Caffeine | | | |  |
| 3-back Error % | 11.26 | 6.49 | [8.84, 13.69] |  |
| 3-back RT | 714.19 | 208.50 | [636.34, 792.05] |  |
| 2-back Error % | 4.37 | 3.87 | [2.93, 5.82] |  |
| 2-back RT | 615.66 | 166.00 | [553.67, 677.65] |  |
| 1-back Error % | 2.23 | 4.28 | [0.65, 3.85] |  |
| 1-back RT | 554.52 | 132.0 | [505.23, 603.81] |  |
| 0-back Error % | 1.50 | 4.28 | [0.00, 0.31] |  |
| 0-back RT | 487.97 | 85.96 | [455.87, 520.06] |  |
| Exercise | | | |  |
| 3-back Error % | 10.49 | 7.25 | [7.78, 13.20] |  |
| 3-back RT | 737.50 | 254.86 | [640.71, 834.29] |  |
| 2-back Error % | 5.88 | 4.97 | [4.02, 7.74] |  |
| 2-back RT | 641.85 | 219.53 | [559.87, 723.82] |  |
| 1-back Error % | 5.60 | 1.81 | [-0.12, 1.23] |  |
| 1-back RT | 543.03 | 140.22 | [490.67, 595.39] |  |
| 0-back Error % | 0.694 | 2.34 | [0.18, 1.57] |  |
| 0-back RT | 488.9 | 78.63 | [459.53, 518.26] |  |

Means, standard deviations, and 95% confidence intervals for n-Back task

| Trial | Non-Caffeine Consumers | | |  |
| --- | --- | --- | --- | --- |
|  | *M* | SD | *95% CI* |  |
| Baseline |  |  |  |  |
| 3-back Error % | 13.06 | 7.00 | [10.40, 15.72] |  |
| 3-back RT | 789.20 | 207.90 | [710.12, 868.28] |  |
| 2-back Error % | 7.23 | 10.60 | [3.20, 11.26] |  |
| 2-back RT | 667.62 | 189.00 | [595.72, 739.52] |  |
| 1-back Error % | 2.93 | 9.096 | [-0.53, 6.389] |  |
| 1-back RT | 551.38 | 162.42 | [489.60, 613.16] |  |
| 0-back Error % | 0.87 | 1.55 | [0.28, 1.46] |  |
| 0-back RT | 473.88 | 59.49 | [451.25, 496.51] |  |
| Caffeine | | | |  |
| 3-back Error % | 10.44 | 8.13 | [7.35, 13.54] |  |
| 3-back RT | 744.93 | 212.36 | [664.15, 825.70] |  |
| 2-back Error % | 4.35 | 9.69 | [0.664, 8.03] |  |
| 2-back RT | 577.34 | 151.62 | [519.66, 635.01] |  |
| 1-back Error % | 3.54 | 8.83 | [0.183, 6.90] |  |
| 1-back RT | 511.56 | 94.87 | [475.47, 547.65] |  |
| 0-back Error % | 1.19 | 2.88 | [0.09, 2.29] |  |
| 0-back RT | 458.82 | 70.98 | [431.82, 485.82] |  |
| Exercise | | | |  |
| 3-back Error % | 10.77 | 7.59 | [7.89, 13.60] |  |
| 3-back RT | 732.44 | 144.71 | [677.40, 787.48] |  |
| 2-back Error % | 5.47 | 8.69 | [2.17, 8.78] |  |
| 2-back RT | 609.61 | 145.28 | [554.35, 664.87] |  |
| 1-back Error % | 2.00 | 8.20 | [-1.00, 6.00] |  |
| 1-back RT | 526.70 | 127.88 | [478.05, 575.33] |  |
| 0-back Error % | 2.00 | 3.60 | [0.00, 3.00] |  |
| 0-back RT | 478.34 | 95.88 | [441.87, 514.81] |  |
